# Supplementary material for: Dynamic context–based updating of object representations in the visual cortex
Source: Sci Adv. 2026 Jan 21;12(4):eadw6726. doi: 10.1126/sciadv.adw6726 (PMC12822648; doi:10.1126/sciadv.adw6726)
Supplement: Supplementary file 1 — Figs. S1 to S7 Tables S1 to S3 [file sciadv.adw6726_sm.pdf]

Science Advances
AAAS

Supplementary Materials for

Dynamic context–based updating of object representations in the visual cortex

Giacomo Aldegheri *et al.*

Corresponding author: Giacomo Aldegheri, giacomo.aldegheri@gmail.com

Sci. Adv. **12**, eadw6726 (2026)
DOI: 10.1126/sciadv.adw6726

This PDF file includes:

Figs. S1 to S7
Tables S1 to S3

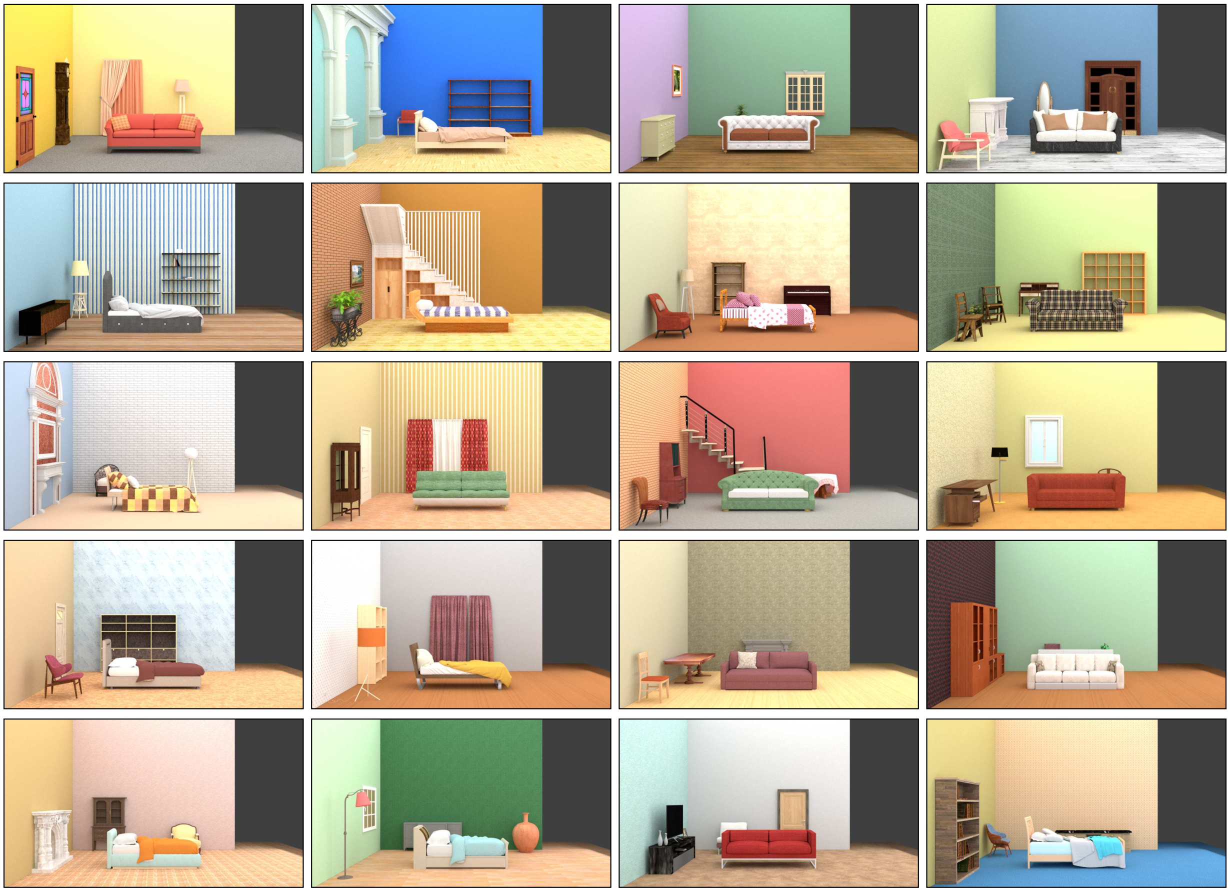

Figure S1. The 20 scene exemplars used in the study.

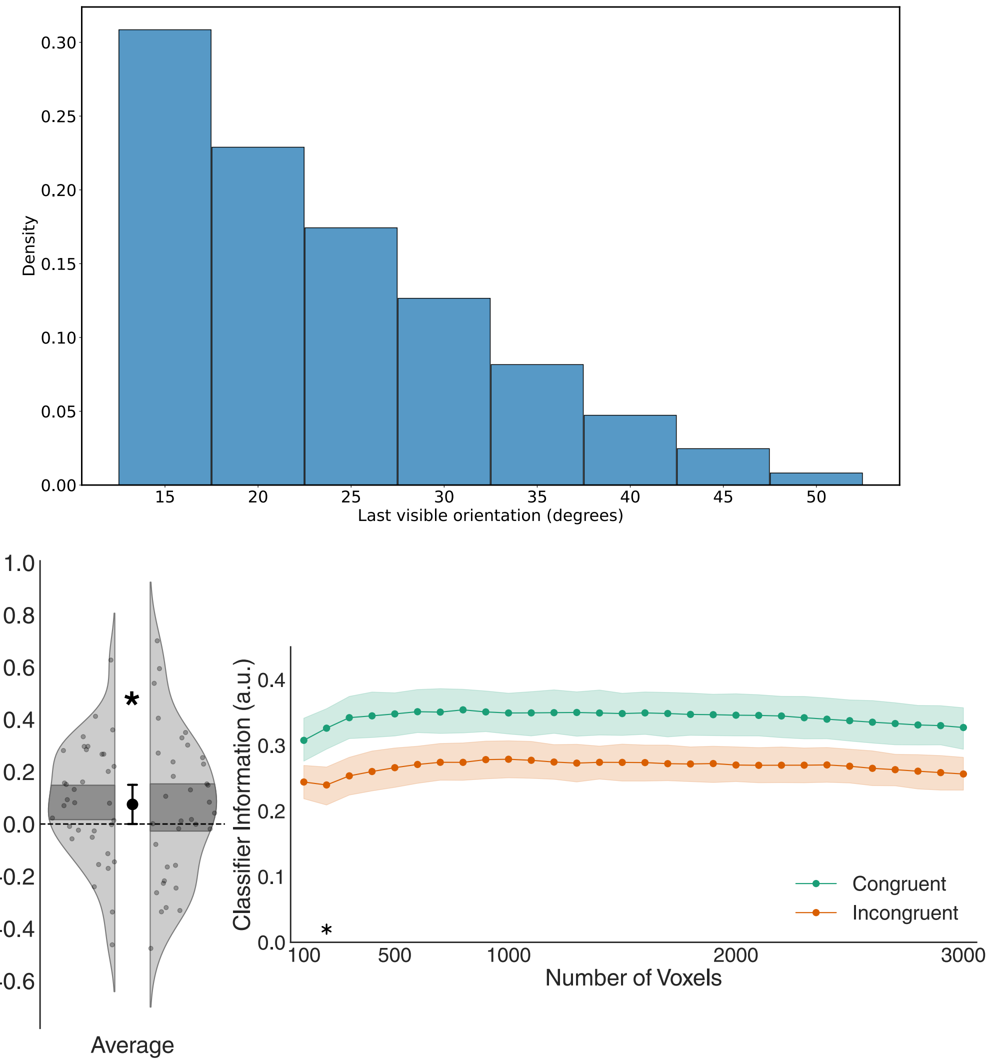

Figure S2. (Top) Distribution of the last visible object orientations, before appearance of the occluder, in the large (i.e., 90°) rotation trials of Experiment 1. In the small (i.e., 30°) rotation trials, this last visible object orientation was always 15°. Thus, in around 30% of the large rotation trials, the last visible orientation of the object was the same as in the small rotation trials, in which case it was impossible to predict the total scene rotation based on this last visible orientation alone. (Bottom) To ensure that the predictive value of the last visible object orientation did not substantially impact the results of Experiment 1, we re-ran the classification analysis of Experiment 1 on EVC, including only the subset of large rotation trials in which the last visible orientation was 15°, thus ensuring that it was perfectly matched (across all trials) with the small rotation condition (for which the last visible orientation was always 15°). As the number of large trials with a last visible orientation of 15° varied randomly from run to run, we had to ensure that the number of trials was balanced between the congruent and incongruent conditions. We thus selected, per each run, the condition (between A90° and B90°, congruent/incongruent) with the smallest number of trials and randomly subsampled trials in the other conditions to match this number. This led to the exclusion of a considerable number of trials. For brevity, this analysis was only run in one decoding direction (training to main task runs), and only selecting subsets of up to 3000 voxels. Despite the substantial reduction in statistical power resulting from the lower number of included trials, this reanalysis of the results of Experiment 1 in EVC replicated the original analysis reported in the main manuscript. * $p < 0.05$

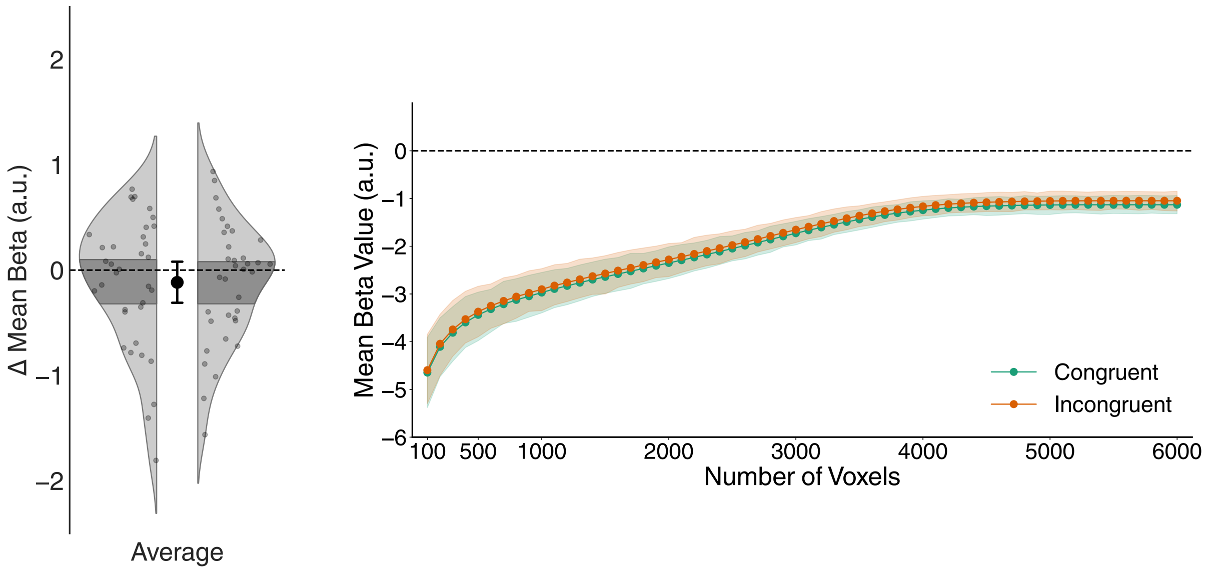

Figure S3. Univariate activation (mean beta value) in EVC for the difference between Congruent and Incongruent trials (left panel) and for Congruent and Incongruent trials separately, across numbers of included voxels (right panel). Univariate activation did not differ in EVC between Congruent and Incongruent trials (and was numerically even higher for Incongruent than Congruent trials), indicating that the increased decodability of object information in Congruent trials did not derive from an increase in overall activation. See **Figure 3B** in the main text for the corresponding multivariate results.

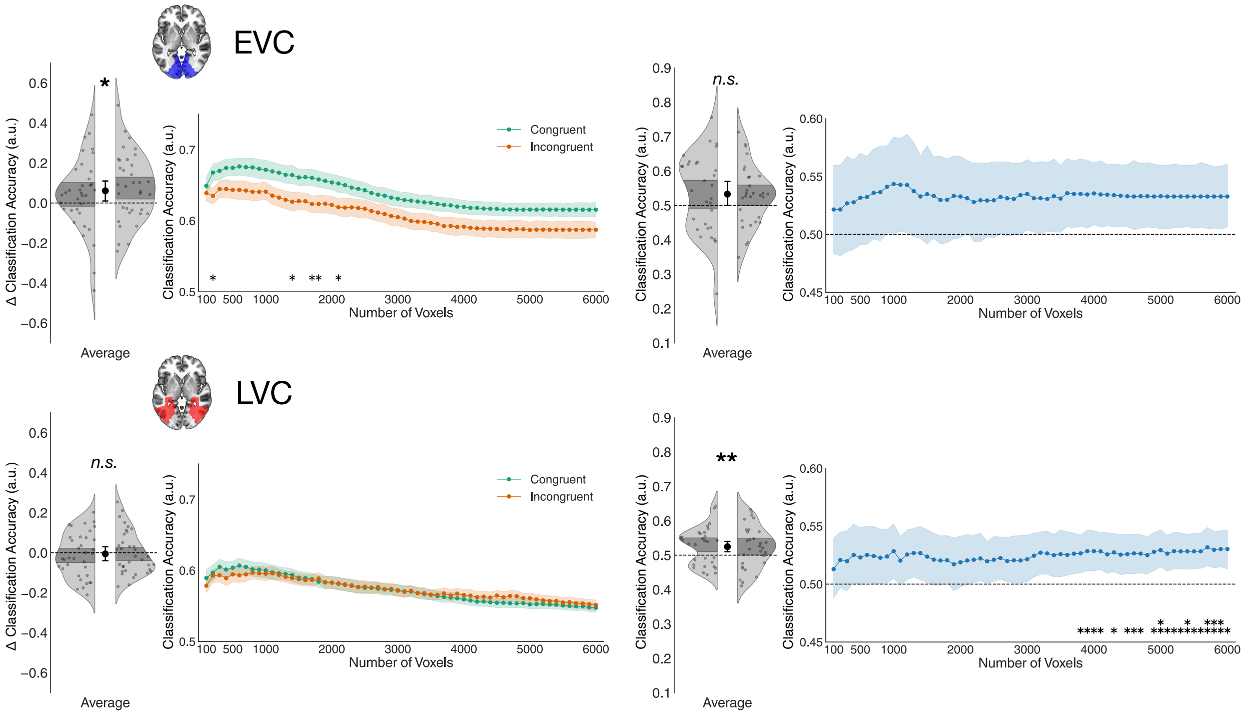

Figure S4. Multivariate decoding results of Experiment 1 (left) and Experiment 2 (right) when using classification accuracy as a measure of information rather than classifier information. See **Figures 3B & 6B** in the main text for the corresponding plots using classifier information.

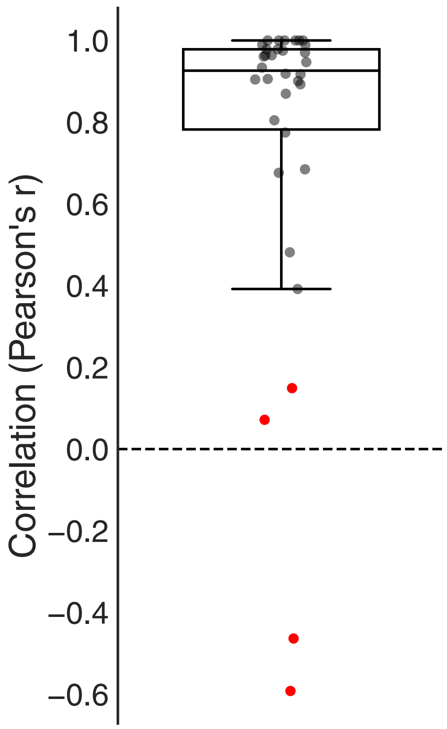

Figure S5. Accuracy in the simple recall task of Experiment 2 for each participant, measured as the Pearson's correlation between participants' estimates and the true number of object reappearances. Points highlighted in red indicates outliers (participants who were more than two inter-quartile ranges away from the first quartile), which were excluded from the analysis. The boxplot indicates first, second (median) and third quartile, and the whiskers are drawn until the farthest point within two inter-quartile ranges from the first quartile, or the minimum among the included participants.

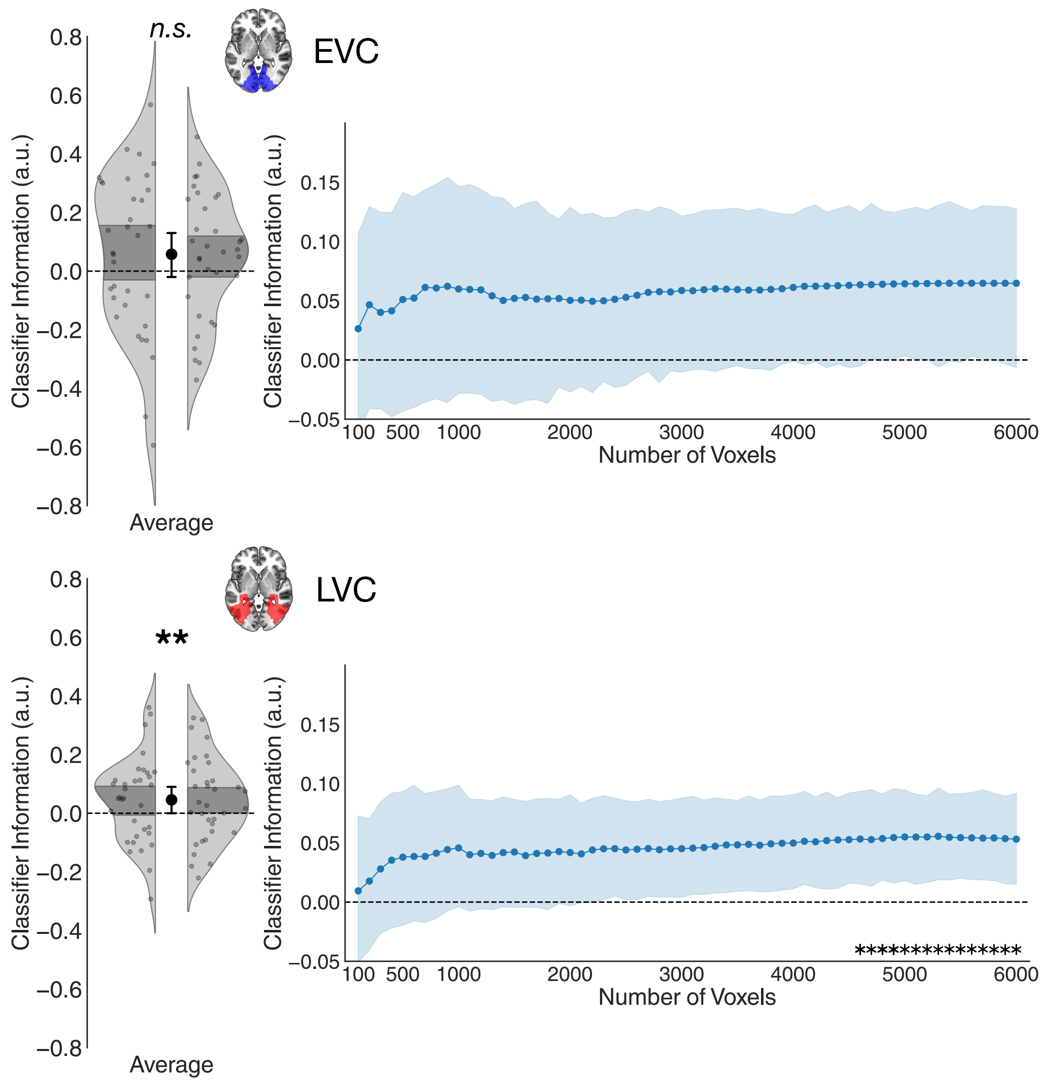

Figure S6. Results of Experiment 2 without any participant exclusions. See **Figure 6B** in the main text for the corresponding results with participant exclusions.

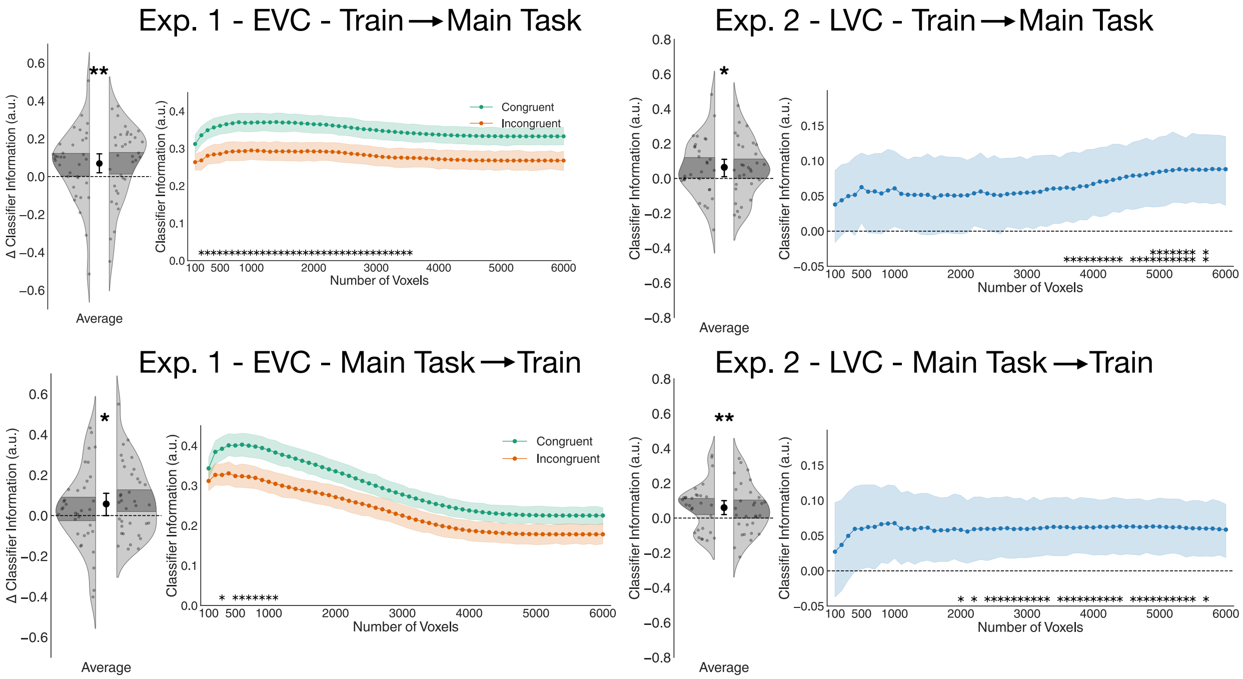

Figure S7. Results for EVC in Experiment 1, and LVC in Experiment 2, separated by decoding direction. The results do not differ in the two directions, although the noise levels are different.

Cluster ID	X	Y	Z	Peak Stat	Cluster Size (mm3)
1	-6.0	-68.0	56.0	5.064	768
1a	-10.0	-62.0	52.0	4.354	
1b	-6.0	-68.0	62.0	3.949	
1c	-14.0	-66.0	62.0	3.601	
2	36.0	-72.0	42.0	4.804	1392
2a	34.0	-56.0	50.0	4.189	
2b	40.0	-64.0	40.0	4.092	
2c	34.0	-58.0	42.0	3.987	
3	-24.0	-66.0	44.0	4.767	816
4	8.0	-70.0	54.0	4.728	840
4a	22.0	-66.0	54.0	4.136	
5	-22.0	-40.0	-8.0	4.590	96
6	28.0	-56.0	36.0	4.388	80
7	16.0	-80.0	-32.0	4.231	88
8	-26.0	-6.0	52.0	4.207	104
9	26.0	-4.0	50.0	4.185	640
9a	26.0	6.0	60.0	4.003	
9b	22.0	12.0	54.0	3.819	
10	-26.0	8.0	58.0	4.164	144
11	-34.0	-54.0	46.0	4.147	184
12	-4.0	-78.0	-24.0	4.132	88
13	-30.0	-76.0	32.0	4.076	192
14	-56.0	-54.0	0.0	4.042	120
15	-26.0	-60.0	54.0	4.025	216
16	20.0	-56.0	24.0	4.001	120
17	-38.0	-44.0	50.0	3.941	440
17a	-40.0	-36.0	48.0	3.932	

18	38.0	10.0	34.0	3.841	120
19	60.0	-50.0	12.0	3.792	192

Table S1. Clusters showing a significantly stronger response for Incongruent relative to Congruent trials in Experiment 1. No clusters were found that showed a significantly stronger response to Congruent than to Incongruent trials.

Cluster ID	X	Y	Z	Peak Stat	Cluster Size (mm3)
1	40.0	-64.0	-4.0	4.559	96
2	50.0	12.0	14.0	4.077	128
3	36.0	-72.0	28.0	4.050	96
4	48.0	-58.0	10.0	3.934	128
5	-44.0	-68.0	6.0	3.842	168
6	54.0	-64.0	-2.0	3.734	136
7	50.0	-72.0	2.0	3.698	80
8	4.0	4.0	56.0	3.607	80
9	46.0	-78.0	2.0	3.551	80

Table S2. Clusters showing a significantly higher correlation on Congruent vs. Incongruent trials with multivariate decoding time courses in EVC (information-activation coupling analysis) in Experiment 1. This analysis revealed clusters in bilateral higher-level visual cortex, and in parietal, premotor and inferior frontal cortex, that were implicated in the enhancement of object information in EVC.

Coordinates: (40, -64, -4)

Name	z-score	Posterior prob.	Func. conn. (r)	Meta-analytic coact. (r)
visual motion	8.56	0.9	0.34	0.37
v5	6.87	0.86	0.38	0.42
motion	6.69	0.78	0.42	0.4
mt	6.15	0.83	0.4	0.43
visual	5.65	0.67	0.67	0.58
occipital	5.63	0.7	0.62	0.52
fusiform	5.16	0.71	0.38	0.34
objects	4.51	0.71	0.42	0.41
occipito temporal	4.28	0.78	0.36	0.36
object	4.16	0.69	0.41	0.38

Coordinates: (50, 12, 14)

Name	z-score	Posterior prob.	Func. conn. (r)	Meta-analytic coact. (r)
inferior frontal	7.08	0.68	0.24	0.24
premotor	6.94	0.71	0.26	0.42
imitation	6.7	0.84	0.11	0.15
handed	6.15	0.75	0.04	0.18
broca	5.64	0.76	0.11	0.13
ventral premotor	5.38	0.77	0.2	0.31
basal ganglia	5.27	0.71	0.11	0.2
ganglia	5.24	0.71	0.11	0.2
inferior	5.22	0.63	0.26	0.24
posterior inferior	5.22	0.81	0.09	0.09

Coordinates: (36, -72, 28)

Name	z-score	Posterior prob.	Func. conn. (r)	Meta-analytic coact. (r)
spatial	6.07	0.7	0.39	0.39
parietal occipital	6.05	0.84	0.06	0.06
visuo	5.27	0.8	0.18	0.13
navigation	5.26	0.84	0.2	0.19
parietal	5.15	0.65	0.4	0.44
occipital	5.02	0.67	0.51	0.3
parietal frontal	4.79	0.79	0.19	0.19
relational	4.63	0.82	0.02	0.05
visuo spatial	4.54	0.83	0.1	0.09
lateral occipital	4.53	0.76	0.3	0.19

Table S3. Top 10 terms associated, in the Neurosynth meta-analysis platform (retrieved 25/11/2024), with the peak coordinates of the positively correlated clusters revealed by the information-activation coupling analysis. It can be seen from these terms that the first two clusters are associated with higher-level visual cortex, particularly motion and object processing, while the third is associated with inferior frontal and premotor cortex.
